# Lactylation: the malignant playbook of hepatocellular carcinoma cells and their roadmap to therapy resistance

**DOI:** 10.3389/fimmu.2025.1752404

**Published:** 2026-01-15

**Authors:** Shuang Li, Xiaoyu Lu, Lu Zhang

**Affiliations:** 1 The First School of Clinical Medicine, Zhejiang Chinese Medical University, Hangzhou, Zhejiang, China; 2 Department of Neurology, Taizhou Hospital of Chinese Traditional and Western Medicine, Taizhou, China; 3 Department of Gastroenterology, Zhejiang Chinese Medical University First Affiliated Hospital (Zhejiang Provincial Hospital of Traditional Chinese Medicine), Hangzhou, Zhejiang, China

**Keywords:** cellular proliferation, hepatocellular carcinoma, lactylation, therapeutic resistance, tumor microenvironment

## Abstract

Hepatocellular carcinoma (HCC) is a significant global health issue, characterized by a high mortality rate closely linked to treatment resistance and late-stage diagnosis. Lactylation, a distinctive post-translational modification linking cellular metabolism and epigenetics, has recently attracted significant attention due to its potential role in HCC. This review aims to systematically elucidate how lactylation facilitates the malignant progression of HCC through many mechanisms, including regulation of cell proliferation, invasion and metastasis, immune response, and stem cell properties. It examines its role in immunotherapy and resistance to targeted therapy in greater depth. The essay succinctly evaluates the therapeutic promise and translational challenges of targeting lactylation, offering a resource for future study.

## 1 Introduction

Hepatocellular carcinoma (HCC) constitutes a significant global health concern, with its prevalence consistently increasing worldwide (1). In 2022, there were 865,269 new cases and 757,948 fatalities due to HCC worldwide, rendering it the sixth most prevalent malignant neoplasm and the third most common cause of cancer-related mortality globally (2). Surgical excision is presently the most effective treatment for HCC. Nevertheless, owing to the absence of discernible early signs, more than fifty percent of patients present at an advanced stage, forfeiting the chance for curative surgery. In patients with advanced or unresectable hepatocellular carcinoma, pharmacological treatments (including targeted medicines and immunotherapy) can extend survival and manage disease progression (3). The considerable variety of HCC results in substantial variability in therapeutic success, with medication resistance becoming a key obstacle that restricts clinical benefit. Consequently, investigating the molecular foundations of resistance and formulating innovative therapeutic approaches is imperative.

In recent years, the interaction between tumor metabolism and epigenetics has attracted considerable interest, with the identification of lactate metabolism providing a new insight into the malignant course of HCC. Lactate was historically considered a byproduct of glycolysis. The lactate shuttle theory has reinterpreted lactate’s role as both a dual fuel and a signaling molecule that coordinates systemic metabolism (4, 5). In 2019, Zhang et al. elucidated that lactate serves as a precursor for histone lysine lactylation, thereby establishing a direct connection between metabolic conditions and epigenetic control (6). Recent studies demonstrate that lactylation significantly contributes to tumor metabolic reprogramming, facilitating malignant growth and treatment resistance in various malignancies through the regulation of gene expression and cellular plasticity (7–9). This review seeks to clarify the fundamental function and molecular mechanisms of lactylation in the malignant behavior and treatment resistance of HCC, and to examine its potential for therapeutic application in targeted therapy.

## 2 Metabolism of lactate and lactylation in neoplasms

Tumor cells display substantial metabolic disparities compared to normal cells, primarily metabolizing glucose to lactic acid even in the presence of oxygen, rather than completely oxidizing it—a phenomenon referred to as the Warburg effect (10). In a traditional cellular context, glucose is first oxidized to pyruvate in the cytoplasm before entering the mitochondria and the tricarboxylic acid (TCA) cycle. This method completely oxidizes glucose to carbon dioxide, optimizing ATP synthesis and inhibiting lactate formation (11). The oxidation of one glucose molecule releases more than 36 ATP molecules. Glycolysis, nonetheless, releases only 2 ATP molecules (12). What accounts for the preference of tumor cells for this inefficient metabolic pathway? Previous studies have demonstrated that glycolysis provides a simpler mechanism for ATP generation, releasing ATP more rapidly than oxidative phosphorylation. Additionally, the vascular distribution and shape within tumor tissues frequently lead to insufficient oxygen delivery. Concurrently, glycolytic intermediates—such as pentose phosphate, glycerol, and acetyl-CoA—function as vital precursors for the synthesis of cellular constituents, including nucleic acids, lipids, and proteins. Opting for glycolytic metabolism allows tumor cells to maintain robust growth even in hypoxic environments (13–17). The Warburg effect exemplifies an adaptive mechanism that enables tumors to maintain rapid growth in challenging conditions.

Nonetheless, vigorous glycolysis leads to significant lactate accumulation. Tumor cells depend on monocarboxylate transporters (MCTs) to export lactate from the cell (18). The exported lactate is subsequently incorporated into a systemic metabolic network. Glycolytic cells, typically found in hypoxic regions, secrete lactate and are linked to respiratory cells, commonly situated near blood vessels, that specialize in oxidative phosphorylation. The latter effectively oxidizes the lactate generated by the former as a fuel, facilitating the recycling of energy and carbon sources within the tumor (16, 19). Additionally, lactate serves as a signaling molecule, modulating critical pathways, including PI3K/AKT/mTOR and Wnt/β-catenin, to enhance tumor proliferation (20).

**Figure 1:**
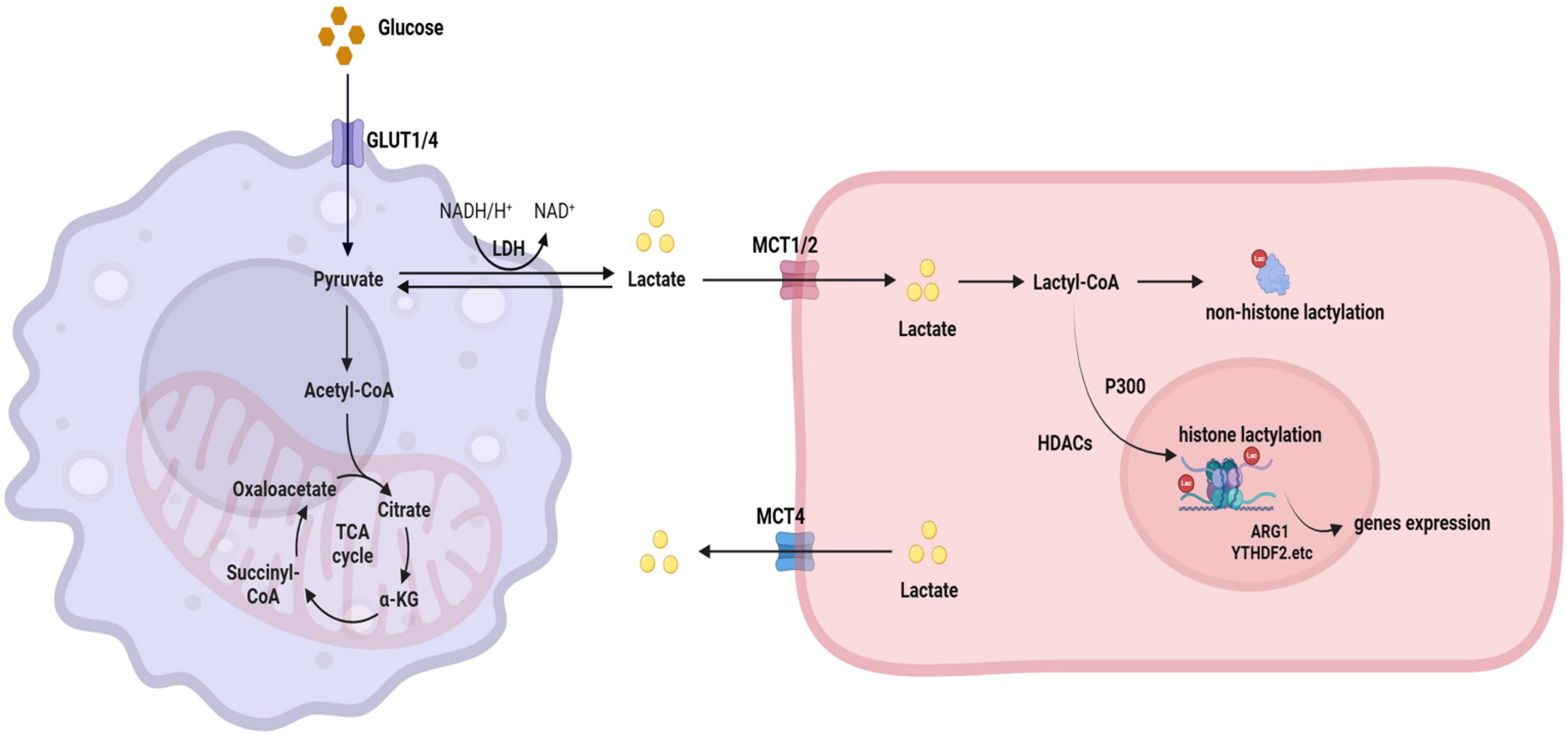
Schematic diagram of lactate metabolism and lactylation in neoplasms. Glucose enters the cell via glucose transporter 1/4 (GLUT1/4) and is converted to pyruvate through glycolysis. Pyruvate is subsequently reduced to lactate, a reaction catalyzed by lactate dehydrogenase (LDH). Lactate can either be exported through monocarboxylate transporter 4 (MCT4), contributing to microenvironmental acidification, or be intracellularly converted to lactyl-CoA, which serves as a direct substrate for the lactylation modification of non-histone proteins. Alternatively, lactate can be transported by monocarboxylate transporters 1/2 (MCT1/2) and utilized by the histone acetyltransferase p300 to catalyze histone lactylation. This lactylation constitutes a dynamically reversible modification regulated by histone deacetylases (HDACs), and can recruit reader proteins such as YTHDF2 to modulate gene expression. Created with BioRender.com.

Finally, the continuous release of lactate by tumor cells leads to overall acidification of the tumor microenvironment (TME) (18). The acidic microenvironment facilitates the development of an immunosuppressive state and serves as a substrate for lactate conversion (6). Therefore, although the Warburg effect does not directly contribute to lactylation, it acts as the principal source of lactate. Lysine lactyltransferase catalyzes the covalent attachment of lactate molecules to lysine residues on target proteins, resulting in the formation of lactylated residues (21). Simultaneously, demodifying enzymes (including HDAC1–3 and SIRT1-3) facilitate the removal of lactylation to preserve dynamic equilibrium (22, 23). The interaction between these two enzyme groups precisely regulates intracellular lactylation levels, consequently affecting essential biological pathways such as cellular metabolism, gene expression, and DNA repair (21). This complex regulatory network ultimately enables cancer cells to adapt to environmental stress and develop treatment resistance (**Figure 1**).

## 3 The impact of lactylation on proteins

Lactylation in tumor biology plays wide, context-dependent regulatory roles, with its specific actions contingent on the altered substrate and cellular environment. Histones are the most thoroughly examined substrates for lactylation, with their modifications directly affecting chromatin architecture and transcriptional activity (6). Research indicates that lactylation at histone H3K18, H3K23, and H4K12 can facilitate tumor growth by altering chromatin accessibility and enhancing transcription in the promoter regions of oncogenes such as TTK and BUB1B (24). Histone H3 lysine 18 lactylation (H3K18la) demonstrates elevated expression in various malignancies, including breast, lung, and colorectal cancers, and is highly correlated with adverse patient prognosis, metastasis, and medication resistance (24–30) (see **Table 1**). The findings across several cancer types offer essential insights and theoretical frameworks for understanding the potential analogous significance of this alteration in HCC, underscoring its potential as a universal tumor biomarker and a prospective therapeutic target.

**Table 1:** Mechanisms of h3K18 lactylation in various cancers.

Cancer types	Lactylation sites	Target/gene	Function	Citation
Pancreatic Ductal Adenocarcinoma	H3K18	TTK、BUB1B	Accelerates cell cycle progression and mitosis	(24)
Breast Cancer	H3K18	PPARD	Promotes proliferation and inhibits apoptosis, activating pro-survival signaling	(25)
Lung Cancer	H3K18	MYC、PD-L1、LDHA、LDHB	Facilitates immune evasion	(26)
Colorectal Cancer	H3K18	MTETTL1	Facilitates immune evasion	(27)
Bladder Cancer	H3K18	LCN2、YY1, YBX1	Promotes cell proliferation, migration, metastasis, and cisplatin resistance	(28, 29)
Eye Melanoma	H3K18	YTHDF2、PER1、TP53	Linking histone modifications with RNA m^6^A modifications to enhance tumor proliferation, migration, and *in vivo* growth	(30)

Lactylation substantially alters non-histone proteins, including metabolic enzymes, transcriptional regulators, and signaling molecules, as well as histones. This non-histone lactylation affects tumor progression through various mechanisms: it modifies intracellular metabolite composition by altering key metabolic enzymes such as LDH and PK, which indirectly influences the activity and substrate availability of other epigenetic modification enzymes (31–33); and it integrates as signaling nodes into essential pathway networks like TGF-β/Smad2 and MAPK, directly regulating fundamental cellular functions including proliferation, differentiation, and survival (34–37). In contrast to histone lactylation, which predominantly affects gene transcription, non-histone lactylation demonstrates greater functional diversity, encompassing processes such as metabolic regulation and signal transduction, thereby broadening the implications of lactylation.

## 4 The pivotal role of lactylation in the malignant advancement of hepatocellular carcinoma

**Figure 2:**
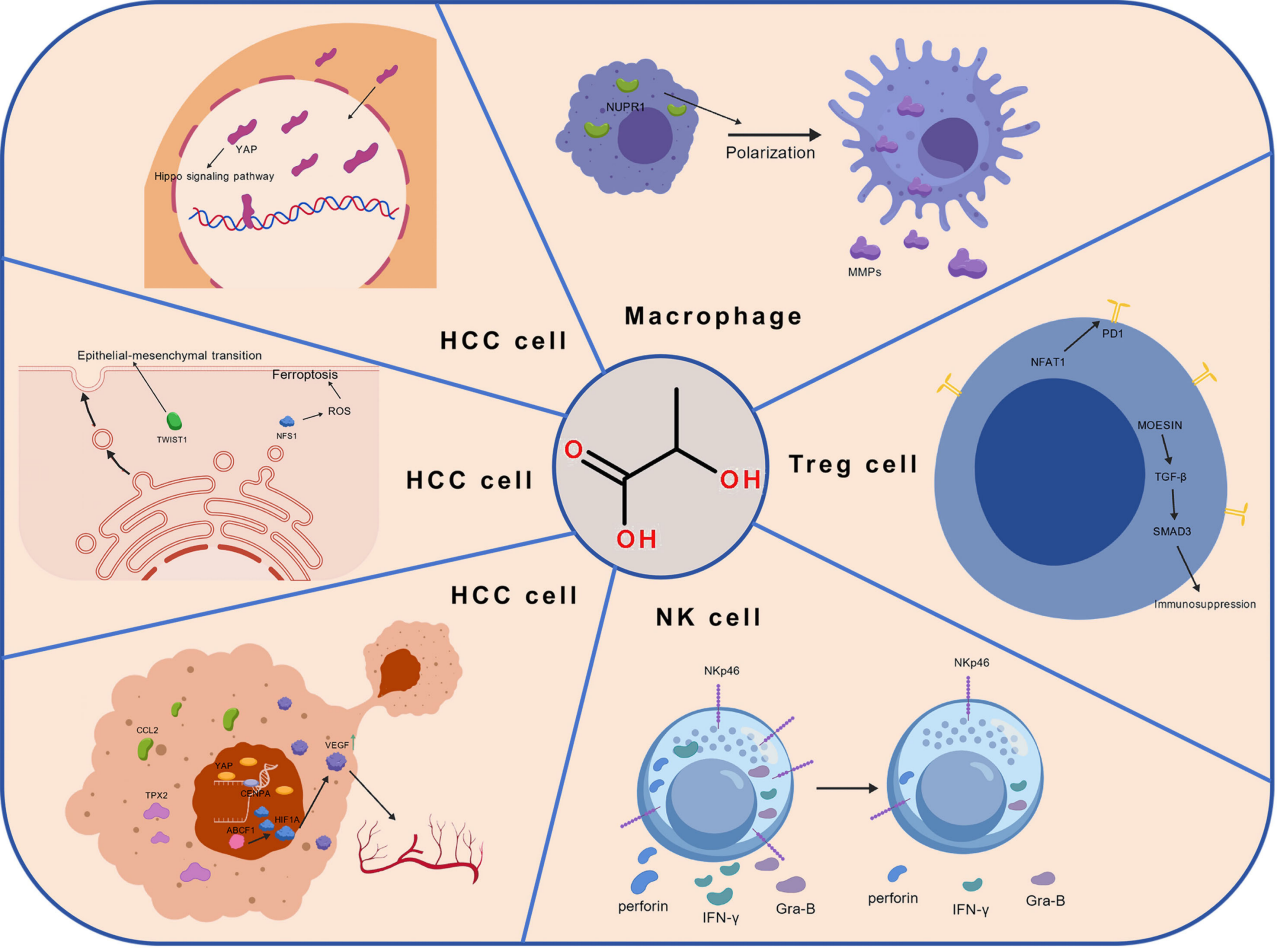
The multidimensional regulatory network of lactylation in the malignant progression of hepatocellular carcinoma. This figure systematically illustrates the core tumor-promoting mechanisms of lactylation in hepatocellular carcinoma (HCC). Beginning with lactate accumulation driven by metabolic reprogramming, lactylation serves as a hub to simultaneously drive malignant progression through multiple pathways: 1) Promoting proliferation by stabilizing Cyclin E2 and activating YAP/mTOR signaling to accelerate the cell cycle; 2) Enhancing metastasis by activating TWIST1 to induce epithelial-mesenchymal transition (EMT), modifying Rab7A to promote pro-metastatic exosome release, and upregulating NFS1 to confer ferroptosis resistance; 3) Mediating immune evasion by suppressing CD8^+^ T and NK cell function and driving macrophage M2 polarization; 4) Stimulating angiogenesis via endothelial histone lactylation and HIF-1a stabilization to upregulate VEGF. Created with BioGDP.com.

The aforementioned content clarifies the diverse regulatory roles of lactylation in transcription, metabolism, and signaling pathways, highlighting its significant regulatory capacity. In the intricate, multifactorial, and multistage development of HCC, this possibility establishes lactylation as a crucial regulatory center. Recent studies increasingly demonstrate that lactylation serves as a fundamental link between metabolism and epigenetics, playing a crucial role in the malignant progression of HCC (**Figure 2**).

### 4.1 Lactylation drives proliferative activity in hepatocellular carcinoma cells

Lactylation-induced differentiation propels malignant growth in hepatocellular carcinoma by influencing cell cycle mechanisms and altering transcriptional regulatory networks. Lactylation-induced differentiation operates as a “dual engine” inside the cell cycle. It not only triggers the G1/S transition by stabilizing cyclin E2 (CCNE2) but also augments the TPX2/Aurora pathway to guarantee effective mitosis (38, 39). These actions interact with the chromatin milieu established by histone H3K56 lactylation, which promotes cyclin gene expression, to provide comprehensive regulation of the entire cell cycle process (40).

Research indicates that the lactylation of the ABCF1 protein at the transcriptional level activates the KDM3A/H3K9me2/HIF-α signaling pathway, facilitating tumor adaptation to hypoxic microenvironments (41); additionally, the lactylation of the centromeric protein CENPA augments its function as a transcriptional co-activator, working in concert with the transcription factor YY1 to enhance the expression of proliferation-related genes such as CCND1 and NRP2 (42). Lactylation also regulates the Hippo pathway effector protein YAP. YAP lactylation impairs its interaction with the export protein CRM1, resulting in atypical nuclear retention of YAP. Conversely, it inhibits YAP phosphorylation, thereby further facilitating its activation and jointly augmenting the malignant character of HCC (43, 44).

mTOR serves as a pivotal regulator of cellular growth and metabolism. ChIP-seq research reveals that mTOR directly associates with regulatory areas of several Pol II-transcribed genes in mouse hepatocellular carcinoma cells. Throughout mouse growth and development, the mTOR signaling pathway directly influences glucose homeostasis by facilitating glycolysis and lactate production, thereby serving as a principal driver of the Warburg effect in HCC (45, 46). Lactylation enhances proliferative signals by regulating the transcriptional activity of mTOR downstream targets, including Cyclin D1 and c-Myc, thereby creating a feedback loop: mTOR-driven glycolysis elevates lactate levels, which facilitates protein lactylation, and lactylation subsequently boosts proliferative signaling. This cycle sustainably exacerbates the aggressive development of HCC cells. Lactylation serves as a pivotal metabolic junction connecting the Warburg effect to the malignant phenotype of HCC (47, 48). Lactylation effectively converts metabolic signals into proliferative outcomes by simultaneously controlling essential cell-cycle proteins, restructuring complex transcriptional networks, and incorporating mTOR-mediated metabolic regulation.

### 4.2 Lactylation augments the invasive and metastatic capabilities of hepatocellular carcinoma

The elevated mortality of HCC is intricately linked to its formidable invasive and metastatic potential. Epithelial-mesenchymal transition (EMT) constitutes a pivotal phase in the invasion and metastasis of HCC. In the TME of HCC, significant lactate buildup activates the lactylation-responsive transcription factor TWIST1, thereby increasing its nuclear translocation efficiency and transcriptional activity. This subsequently activates the EMT pathway, allowing cancer cells to adopt a mesenchymal phenotype and thereby enhancing their invasive and metastatic capabilities (49). This procedure unequivocally illustrates that lactylation serves as a fundamental molecular foundation for commencing the metastatic program in cancer cells. Moreover, metastasis is not a solitary biological phenomenon; it entails interaction among cell populations. Lactate promotes the lactylation of the multivesicular body transport-associated protein Rab7A. This impedes its GTPase activity, resulting in the fusion of multivesicular bodies with the plasma membrane and facilitating the release of tumor exosomes. These exosomes function as molecular messengers, conveying extracellular matrix remodeling proteins to remote organs, thus establishing a pre-metastatic milieu for HCC cell colonization (50). This clearly illustrates the essential function of lactylation in facilitating the interaction between tumors and their microenvironments.

Lactylation not only regulates metastasis initiation and cellular communication but also provides survival advantages to cancer cells during metastasis via epigenetic regulation and modulation of signaling pathways. Under stress conditions, H3K18la markedly increases HCC cells’ resistance to ferroptosis by upregulating the iron-sulfur cluster assembly factor NFS1, thereby facilitating their survival during metastasis (51). Simultaneously, lactylation of insulin receptor substrate 1 (IRS1) enhances its protein stability and initiates downstream pro-survival signaling, so ensuring prolonged nutritional support for the colonization and expansion of metastatic sites (52). These results collectively demonstrate that lactylation markedly improves HCC metastatic efficiency via a tripartite synergistic mechanism: initiation (EMT), amplification (exosomes), and consolidation (survival). A comprehensive examination of the particulars of this regulatory mechanism enhances our understanding of the molecular processes underlying HCC metastasis and provides essential theoretical support for the future formulation of multi-target combination therapies targeting HCC metastasis.

### 4.3 Lactylation promotes immunological evasion through the regulation of immune cells

Tumor progression is contingent not only on the malignant characteristics of cancer cells but also, significantly, on the support provided by their TME. The TME harbors a plethora of immune cells that can facilitate antitumor immune responses while also being exploited by tumors, therefore advancing disease development and affecting therapy efficacy (53–55). Lactate exerts functional inhibition on CD8^+^ T cells and natural killer (NK) cells. It suppresses CD8^+^ T cell proliferation and cytokine release by modulating nicotinamide adenine dinucleotide (NAD) and NADH levels, thereby markedly diminishing their cytotoxicity (56). Concurrently, it disrupts NK cells by diminishing the expression of the surface activation receptor NKp46 and decreasing the release of crucial anticancer factors, including interferon-γ (IFN-γ), perforin, and granzyme B, thereby impairing innate immune surveillance functions (57, 58).

Lactate and lactylation actively promote the differentiation, polarization, and activation of immune cell populations to reinforce immunosuppression. In macrophages, p300-mediated histone lactylation directly facilitates transcriptional reprogramming by enhancing the promoters of typical M2-associated genes, such as arginase (Arg1) (6). This method establishes a positive feedback loop with the splicing factor SRSF1, synergistically augmenting M2 polarization and further inhibiting CD8^+^ T cell activity (59). M2-polarized macrophages release immunosuppressive substances, including transforming growth factor-β (TGF-β) and interleukin-10 (IL-10), which further impair antitumor immunity and facilitate tumor growth and spread (60). Nonetheless, the effects of lactate on immune cells are not invariably immunosuppressive. Research indicates that, in particular, CD4^+^ T cell subsets, lactate metabolism facilitates Th1 cell development and augments antitumor immunity through JAML^+^ CD8^+^ T cells (61). This discovery underscores the bifunctional immunoregulatory role of lactate, indicating that subsequent research should concentrate on targeted intervention measures. These results illustrate that lactate and lactylation facilitate HCC immune evasion by modulating immune cell function and provide a biological foundation for the development of primary or secondary resistance to immunotherapy.

### 4.4 Lactylation enhances angiogenesis in hepatocellular carcinoma

HCC is a highly vascular tumor whose rapid proliferation and spread are fundamentally reliant on angiogenesis. These neovessels provide crucial nutrients for tumor proliferation and function as critical pathways for cancer cell spread. Recent studies indicate that lactate accumulation in TME and the resulting lactylation are significant contributors to angiogenesis in HCC. Studies demonstrate that lactate directly increases lactylation at H3K9 and H3K18 sites in endothelial cells, thereby augmenting the transcriptional activity of angiogenic genes and facilitating pathological angiogenesis (62, 63). Lactate not only directly affects endothelial cells but also modulates the expression of critical angiogenic factors via indirect mechanisms. Lactate stabilizes and activates hypoxia-inducible factor-1α (HIF-1α), thereby triggering the expression of essential angiogenic factors like VEGF. This mechanism significantly stimulates endothelial cell proliferation, migration, and the development of vascular lumens (41, 64). A high-lactate environment simultaneously induces tumor-associated macrophages to adopt a pro-angiogenic phenotype. These cells enhance intravascular signaling within tumors by secreting numerous active factors, including angiopoietins and matrix metalloproteinases (65–67).

A recent study has uncovered a unique method by which lactate collaborates with other regulatory variables to affect angiogenesis in HCC. Histone lactylation facilitates HCC angiogenesis via the c-Myc/GP73/STAT3 system, regarded as a complex regulatory network focused on GP73 and including other pathways, including lactate metabolism, histone modification, c-MYC, and JAK2/STAT3 (68). This discovery enhances the understanding of lactate and lactylation’s function in modulating HCC angiogenesis.

### 4.5 Lactylation affects liver cancer stem cells

Liver cancer stem cells (LCSCs) are pivotal in HCC progression, facilitating primary tumor growth and distant metastasis while substantially reducing the effectiveness of anti-tumor therapy. A research team from Hubei University of Chinese Medicine initially investigated lactate metabolism and lactylation levels between HCC cells and LCSCs, demonstrating that HCC cells display elevated glycolytic activity and lactylation levels. The scientists also found that ALDOA lactylation modulates its interaction with DDX17, thereby affecting DDX17 nuclear translocation and enhancing stemness maintenance in LCSCs (69). Functional investigations demonstrated that the creation of an ALDOA delactylation mutant markedly impedes tumor cell proliferation, metabolism, and tumorigenic potential, highlighting the essential significance of this alteration. Furthermore, pharmacological intervention studies corroborate this mechanism: demethylzeylasteral was shown to significantly reduce global histone lactylation levels, thereby reducing LCSCs stemness and tumorigenic potential (38). Collectively, our data underscore the prospective potential of targeting lactylation as an innovative approach to surmount therapeutic resistance and tumor recurrence associated with LCSCs.

## 5 Fundamental processes of lactylation in the resistance to therapy in hepatocellular carcinoma

The emergence of medication resistance is a significant obstacle hindering clinical effectiveness in advanced HCC patients. Lactylation, a recently identified post-translational modification, has been extensively implicated in mechanisms of treatment resistance in HCC across multiple studies. This discovery may identify novel therapeutic targets for the treatment of HCC. The subsequent sections will methodically examine the fundamental molecular pathways by which lactylation facilitates drug resistance in targeted therapy, immunotherapy, and chemotherapy approaches.

### 5.1 Resistance to targeted therapy mediated by lactylation

Targeted therapy is the principal clinical intervention for advanced HCC (70, 71). Tyrosine kinase inhibitors (TKIs), including sorafenib and lenvatinib, play a crucial role in therapy by obstructing signaling pathways associated with tumor development, reducing angiogenesis, and triggering apoptosis (72). Nonetheless, the therapeutic effectiveness of these medications is frequently significantly constrained by the emergence of resistance. Recent studies have demonstrated that resistance mechanisms are modulated by lactylation at various levels (73). We shall succinctly delineate the particular mechanisms involved.

#### 5.1.1 Stimulates antioxidant and pro-survival pathways

Lactylation may reduce medication efficacy by activating antioxidant and pro-survival signaling pathways. Central to this process, metabolic stress caused by TKIs exacerbates glycolysis in tumor cells, resulting in lactate buildup. This induces lactylation of essential proteins, hence triggering the cellular self-protection mechanism focused on nuclear factor E2-related factor 2 (NRF2). Lactylation in regorafenib resistance amplifies NRF2-mediated antioxidant responses and inhibits ferroptosis through the ZNF207/PRDX1/NRF2 pathway, thereby increasing the survival ability of HCC cells (74). In a lenvatinib-resistant model, lactate-induced lactylation of IGF2BP3 modifies serine metabolism pathways by increasing the expression of PCK2 and NRF2, thereby augmenting the oxidative defenses of HCC cells and ultimately fostering drug tolerance (75); lactylation of HECTD2 diminishes the therapeutic efficiency of lenvatinib via an analogous antioxidant mechanism by stimulating the KEAP1/NRF2 signaling pathway (76). These results collectively suggest that targeting the lactylation-NRF2 axis may be a highly effective technique for overcoming resistance to targeted therapy in HCC.

#### 5.1.2 Initiates pro-angiogenic signaling

The TKIs employ an additional critical mechanism by inhibiting genes such as VEGF to block the formation of new blood vessels in tumor tissues. The lactylation of HIF-1α increases its stability and activity, hence facilitating the production of downstream genes such as VEGF and encouraging angiogenesis. Research demonstrates that the inhibition of HIF-1α lactylation in HCC effectively obstructs its vasculogenesis signaling and reinstates the sensitivity of tumor cells to ferroptosis (77). This may signify a principal mechanism contributing to resistance against TKIs such as sorafenib.

### 5.2 Immunotherapy resistance mediated by lactylation

Immunotherapy constitutes a vital therapeutic alternative for advanced HCC. Programmed death-1 (PD-1) inhibitors are fundamental drugs in clinical immunotherapy (78). Nonetheless, its clinical response rate is restricted, as the majority of patients exhibit primary or secondary resistance, hence considerably diminishing the therapeutic efficacy of immunotherapy. Recent research suggests that lactate and lactylation play a crucial role in establishing the immunosuppressive tumor microenvironment, which is a fundamental mechanism contributing to resistance against immunotherapy.

#### 5.2.1 Modifies the action of immunosuppressive cells

Lactylation-induced immune evasion constitutes the biological foundation for immunotherapy resistance, with the functional reprogramming of immunosuppressive cells being pivotal in this mechanism. Regulatory T cells (Tregs), a fundamental immunosuppressive fraction inside the tumor microenvironment, aggressively absorb lactate through MCT1. This absorption facilitates the activation of the NFAT1/PD-1 signaling pathway, resulting in an increase in PD-1^+^ Tregs. Simultaneously, lactylation at the K72 residue of the MOESIN protein augments the TGF-β/SMAD3 signaling pathway, hence compromising Treg-mediated immunosuppressive function (37, 79). In addition to Tregs, lactate metabolism and lactylation also contribute to therapy resistance by altering macrophage activity. Research indicates that H3K18la in macrophages particularly enhances the transcription of nuclear protein 1 (NUPR1). Increased NUPR1 expression not only strengthens the M2-polarized phenotype of tumor-associated macrophages (TAMs) but also directly inhibits T cell activity by activating the PD-L1/SIRPA signaling pathway, thereby reducing the anticancer effectiveness of PD-1 inhibitors (80). Intervention techniques focused on lactate metabolism exhibit synergistic potential with immunotherapy. The MCT1 inhibitor ARC155858 can impede tumor proliferation by modulating Treg activity and enhancing the expression of antitumor cytokines (81); preclinical investigations demonstrate that the combination of the MCT1 inhibitor AZD3965 with anti–PD-1 therapy markedly diminishes lactate concentrations in the tumor microenvironment and reactivates the cytotoxic function of T cells (82). Likewise, the combination of MCT4 inhibitors with PD-1 inhibition has exhibited synergistic antitumor effects in many models (83). This evidence substantiates the significant involvement of lactate and lactylation in immunotherapy resistance and indicates that combinatorial strategies aimed at lactate metabolism may represent a promising avenue for surmounting this resistance.

#### 5.2.2 Regulates the expression and stability of immunological checkpoints

Lactylation can directly influence immunological checkpoint molecules, augmenting tumor cell immune evasion by dual pathways of transcriptional activation and protein stability. Lactate stimulates GPR81 on tumor cell surfaces, triggering the downstream TAZ/TEAD transcriptional cascade to directly enhance PD-L1 gene expression (84). Conversely, in immunological modulation, H3K18la can enhance the production of the dome protein (MVP). MVP, as a molecular scaffold, markedly improves the stability of the PD-L1 protein, inhibiting its degradation and resulting in prolonged high expression of PD-L1 on tumor cell surfaces (85). The highly expressed PD-L1 interacts with PD-1 on T cell surfaces, delivering strong inhibitory signals that effectively suppress T cell antitumor actions. Moreover, lactylation indirectly but significantly hinders immune treatment responses by creating an immunosuppressive milieu characterized by the interplay between metabolism and inflammation. Bioinformatics research indicates that patients with elevated expression of inflammatory response genes demonstrate inferior responses to PD-L1 immunotherapy (86). Aberrant activation of the glycolytic system augments tumor cell immune evasion by synergistically facilitating inflammatory gene expression and lactate buildup (87).

### 5.3 Chemotherapy resistance mediated by lactylation

Chemotherapy resistance is a significant factor contributing to treatment failure in HCC. Cancer cells sometimes demonstrate innate or acquired resistance to many chemotherapeutic agents, hence constraining therapeutic efficacy. Recent studies indicate that lactylation improves tumor cell survival via two primary mechanisms: augmenting DNA damage repair capabilities and facilitating medication efflux.

#### 5.3.1 Augments DNA repair capability

Chemotherapy eradicates tumor cells by causing DNA damage, including strand breaks, base alterations, and crosslinking. Tumor cells can evade treatment by aberrantly activating or augmenting DNA damage response (DDR) mechanisms to preserve genomic integrity, resulting in drug resistance. Recent findings indicate that lactylation directly influences critical proteins in the DDR pathway, augmenting their functionality and complex formation to enhance repair fidelity. A 2024 study presented direct evidence that, within the high-lactate tumor microenvironment, the lactylation of lysine 388 (K388) on NBS1—a crucial element of the MRE11-RAD50-NBS1 (MRN) complex—facilitates complex formation, thereby markedly improving homologous recombination (HR) repair efficiency. Lactylation of lysine 673 on MRE11 similarly augments its DNA binding and end-excision activity (88). Inhibiting lactylation at these specific sites impairs repair function and restores tumor cell sensitivity to DNA-damaging drugs like cisplatin. Inhibiting lactylation at these particular locations compromises repair function and reinstates tumor cell sensitivity to DNA-damaging agents such as cisplatin. Lactylation not only directly influences DNA repair but also indirectly enhances the expression of DNA repair genes through the GPR81 signaling pathway. GPR81 is a crucial protein modulated by lactate. The tumor-promoting effects are linked to many signaling mechanisms. It diminishes IFN-γ expression through the Gβγ/Ca²^+^/calmodulin-dependent protein kinase pathway, so directly undermining the immune system’s assault on malignancies. It upregulates the expression of DNA repair genes such as BRCA1 via the PKC/ERK signaling pathway, thereby augmenting tumor cells’ capacity to repair DNA damage induced by chemotherapeutic agents, resulting in chemotherapy resistance (20, 89–91).

#### 5.3.2 Facilitates drug outflow and lowers intracellular drug concentration

The ATP-binding cassette (ABC) transporter family predominantly harnesses energy from ATP hydrolysis to expel anticancer medicines, markedly decreasing drug concentrations in tumor cells and hence fostering treatment resistance. P-glycoprotein, multidrug resistance-associated protein (MRP), and breast cancer resistance protein (BCRP) function as essential molecules facilitating drug efflux. Recent studies demonstrate that epigenetic changes, specifically lactylation, can modulate the production and function of ABC transporters. Proteomics research indicates that H3K18la augments the transcription of ABCB1 and ABCC1 in colorectal and hepatocellular carcinomas, facilitating the efflux of many medicines, including chemotherapeutic treatments (92, 93). Preclinical experiments further validate that lactylation can directly modulate the expression or activity of genes encoding drug efflux pumps (such as ABC transporters) or those implicated in drug metabolism, consequently diminishing the effective intracellular concentration of drugs like oxaliplatin or hastening their inactivation (94, 95). Moreover, specific histone lactylation facilitates the emergence of drug-resistant phenotypes in hepatocellular carcinoma by enhancing MDR1 (ABCB1) expression (96, 97). Despite the aforementioned evidence suggesting a strong correlation between lactylation and ABC transporters in facilitating chemotherapy resistance, existing research on the specific molecular mechanisms through which lactylation modulates ABC transporter expression and function in hepatocellular carcinoma is still insufficient. Subsequent research should investigate its regulatory networks and translational capabilities.

## 6 Discussion and future perspectives

This comprehensive review elucidates the role of lactylation in the advancement of HCC by modulating many biological processes, such as cell proliferation, invasion and metastasis, immune evasion, and stemness. Additionally, we systematically analyze the principal molecular networks by which lactylation facilitates resistance across three predominant clinical treatment modalities: targeted therapy, immunotherapy, and chemotherapy. The distinct molecular pathways vary among these therapeutic contexts: targeted therapy resistance mainly entails the activation of intrinsic survival mechanisms in tumor cells, immunotherapy resistance centers on the reconfiguration of the immunosuppressive microenvironment, and chemotherapy resistance highlights the augmentation of DNA repair and drug efflux mechanisms. Notwithstanding these unique methods, they originate from a shared metabolic source linked to lactate buildup induced by the Warburg effect. These findings enhance the comprehension of HCC biology and promote lactate-induced lactylation from a basic biological occurrence to a potential treatment target.

Therapeutic approaches aimed at lactylation have now developed into a multifaceted strategic framework. At the level of source control, inhibition of critical glycolytic nodes such as GLUT, HK2, and LDHA, or the application of glucose analogues such as 2-DG to diminish lactate generation, has demonstrated antitumor efficacy and increased sensitivity to standard therapies in preclinical models (98–100). The transport blockade targeting MCT1/4 not only directly suppresses tumor proliferation but also exhibits encouraging synergistic therapeutic potential when used in conjunction with immune checkpoint medications. This method efficiently reduces lactate concentrations in the tumor microenvironment and mitigates immunosuppression. Simultaneously, efforts aimed at regulating lactylation are advancing. Small compounds like β-alanine and magnolol can reinstate tumor suppressor protein activity (e.g., p53) or counteract pro-tumor alterations by regulating the lactylation levels of particular proteins (39, 101). Moreover, integrated approaches focusing on critical proteins such as mTOR and GPR81, in conjunction with lactylation pathways, facilitate multi-target modulation of downstream processes, presenting novel avenues for precision therapy in liver cancer—especially for multidrug-resistant, recurrent, and highly invasive HCC (102). Significantly, addressing metabolic irregularities in HCC, encouraging healthy dietary and lifestyle practices, and mitigating muscle loss can substantially decelerate the malignant progression of HCC and the emergence of drug resistance (103).

Nonetheless, actualizing this promise in a therapeutic context is beset by considerable hurdles. Many current inhibitors, including glycolysis inhibitors, have inadequate target selectivity and limited therapeutic windows. The creation of innovative molecules with improved selectivity and increased safety profiles is a pressing necessity for clinical use. The effects of lactylation demonstrate considerable context reliance. The regulatory impact varies significantly across cell types, microenvironmental conditions, and disease progression phases, requiring intervention strategies that reject a “one-size-fits-all” approach. In light of these challenges, future research should progress collectively in the subsequent directions: Larger cohorts of HCC patients are required to systematically delineate HCC-specific lactylation signatures utilizing proteomics, metabolomics, and further technologies. This will delineate essential functional locations across many subtypes and disease stages, offering direct clinical evidence for target selection. Secondly, investigate the interacting networks involving lactylation, immune cells, cancer-associated fibroblasts, and other matrix components inside drug-resistant microenvironments to identify potential targets for effective combination therapies. Simultaneously, expedite the clinical translation and validation of lactylation-associated prognostic models and biomarkers to enhance patient stratification and individualized treatment. Ultimately, integrated treatments that combine metabolic interventions aimed at lactylation with lifestyle management should be investigated to systematically enhance the tumor microenvironment and assist in delaying disease progression.

In conclusion, investigations into lactylation provide novel perspectives and avenues for the prevention and management of HCC. As our understanding of its molecular mechanisms and functional networks advances, we assert that the close integration of fundamental research and clinical practice will render intervention strategies targeting lactylation essential to comprehensive HCC treatment, thereby offering new avenues to enhance patient outcomes.
